# Supplementary material for: Gold Nanoparticles Inhibit Extravasation of Canine Osteosarcoma Cells in the Ex Ovo Chicken Embryo Chorioallantoic Membrane Model
Source: Int J Mol Sci. 2023 Jun 7;24(12):9858. doi: 10.3390/ijms24129858 (PMC10297960; doi:10.3390/ijms24129858)
Supplement: Supplementary file 1 [file ijms-24-09858-s001.zip › ijms-2399291-supplementary.pdf]

Video S1. OSCA-32 cells circulating within the CAM vessels' bloodstream, immediately after the intravenous injection.

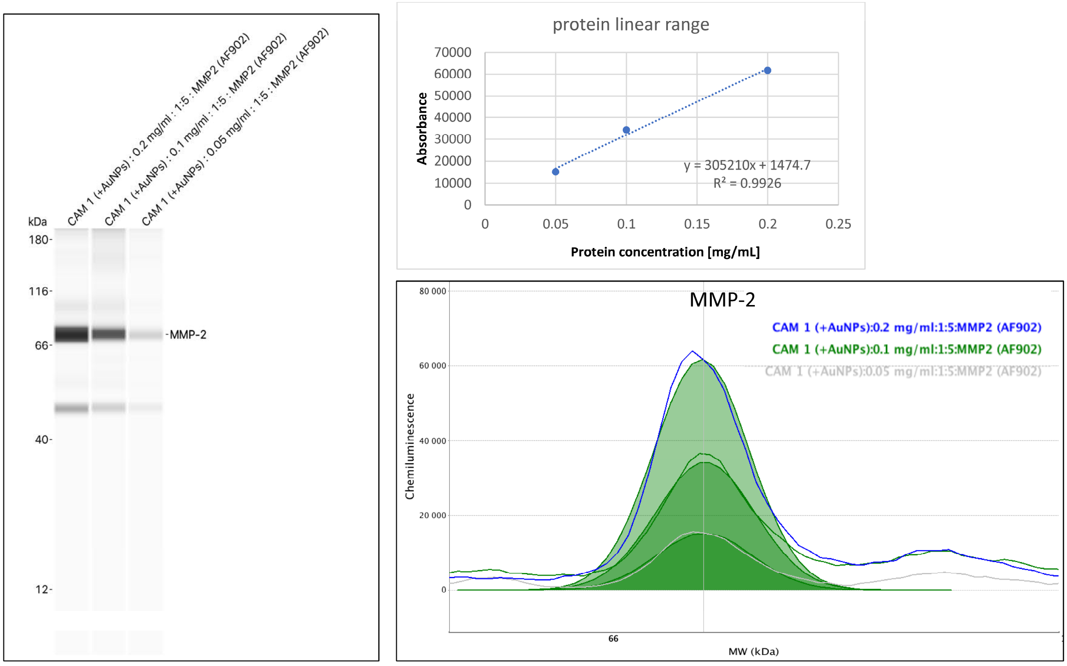

Figure S1. Optimization for automated capillary electrophoretic-based immunoassay method for quantitative MMP-2 expression in the CAM injected with OSCA-8 and OSCA-32 cell lines with or without Au-GSH NPs. Protein linearity.

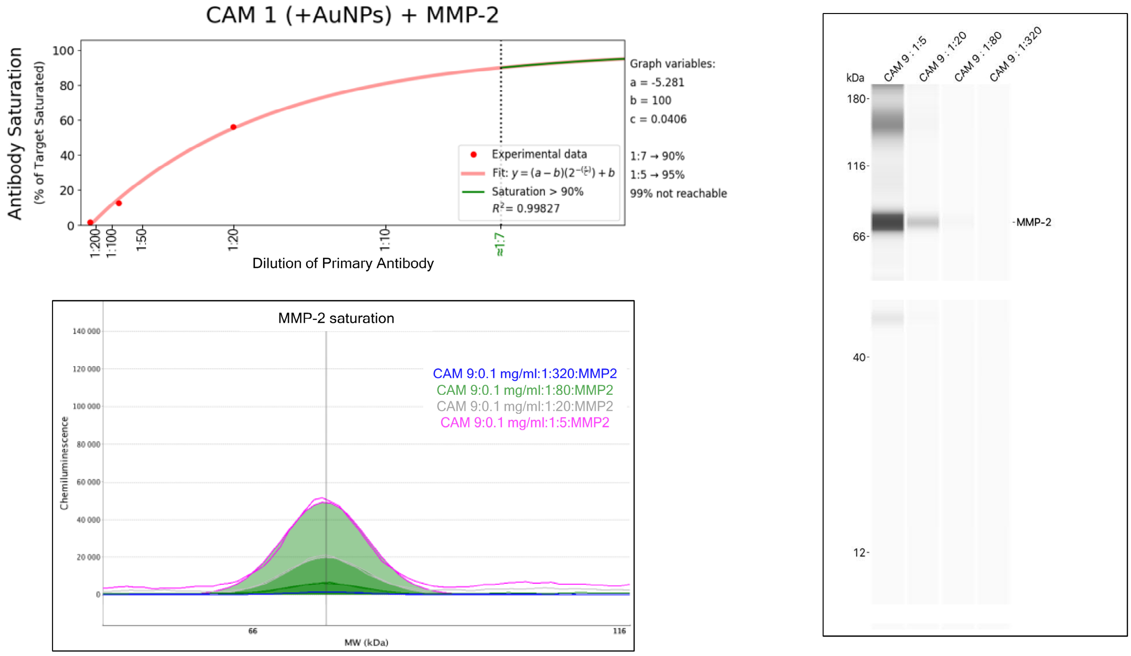

Figure S2. Optimization for automated capillary electrophoretic-based immunoassay method for quantitative MMP-2 expression in the CAM injected with OSCA-8 and OSCA-32 cell lines with or without Au-GSH NPs. Antibody saturation.

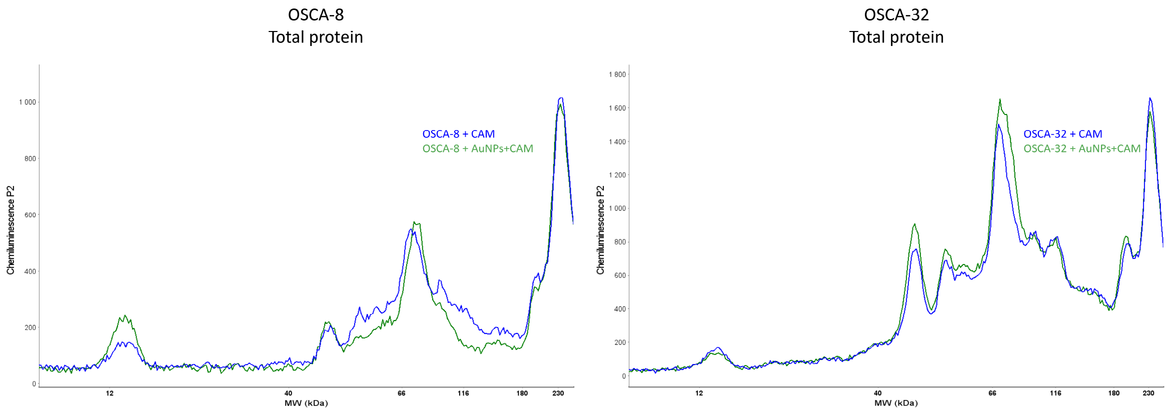

Figure S3. Optimization for automated capillary electrophoretic-based immunoassay method for quantitative MMP-2 expression in the CAM injected with OSCA-8 and OSCA-32 cell lines with or without Au-GSH NPs. Total protein normalization.

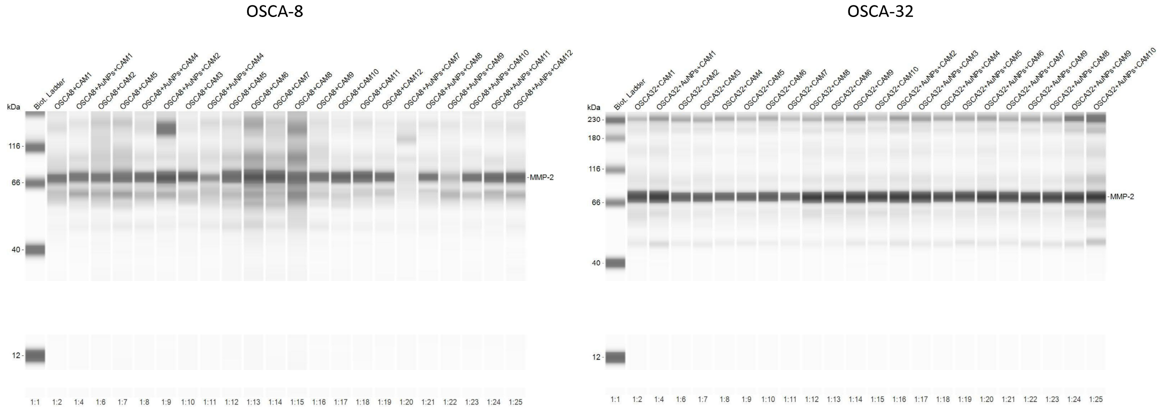

Figure S4. MMP-2 expression.
